# Multifactorial QT Interval Prolongation and Takotsubo Cardiomyopathy

**DOI:** 10.1155/2014/213842

**Published:** 2014-04-14

**Authors:** Michael Gysel, Alexander Crystal, Jules C. Hancox, Michelle Methot, Adrian Baranchuk

**Affiliations:** ^1^Department of Medicine, Kingston General Hospital, Queen's University, Kingston, ON, Canada K7L 3N6; ^2^Department of Medicine, Yale University, Bridgeport Hospital, Bridgeport, CT 06610, USA; ^3^School of Physiology and Pharmacology, University of Bristol, Bristol BS8 1TD, UK; ^4^Cardiac Electrophysiology and Pacing, Kingston General Hospital, Queen's University, Kingston, ON, Canada K7L 2V7

## Abstract

A 71-year-old woman collapsed while working as a grocery store cashier. CPR was performed and an AED revealed torsades de pointes (TdP). She was subsequently defibrillated resulting in restoration of sinus rhythm with a QTc interval of 544 msec. Further evaluation revealed a diagnosis of Takotsubo Cardiomyopathy (TCM) contributing to the development of a multifactorial acquired long QT syndrome (LQTS). The case highlights the role of TCM as a cause of LQTS in the setting of multiple risk factors including old age, female gender, hypokalemia, and treatment with QT prolonging medications. It also highlights the multifactorial nature of acquired LQTS and lends support to growing evidence of an association with TCM.

## 1. Introduction

Acquired long QT syndrome (LQTS) is a disorder of delayed cardiac repolarization that predisposes individuals to a life-threatening tachyarrhythmia known as* torsades de pointes* (TdP). It is often precipitated by the use of QT prolonging medications and the presence of electrolyte disturbances such as hypokalemia and hypomagnesemia. Additional risk factors include old age, female gender, structural heart disease, bradycardia, and the presence of congenital LQTS [1].

Takotsubo Cardiomyopathy (TCM) is a disorder characterized by temporary left ventricular apical ballooning in the absence of significant left main or left anterior descending coronary artery disease. Recent evidence of an association between TCM and acquired LQTS suggests that TCM should be considered amongst its causes [2, 3]. In this report we present a new case of TCM-associated QT interval prolongation and TdP. The case highlights the multifactorial nature of acquired LQTS, the role of TCM in QT prolongation, and the importance of early recognition to ensure appropriate treatment.

## 2. Case Presentation

**Figure 1:**
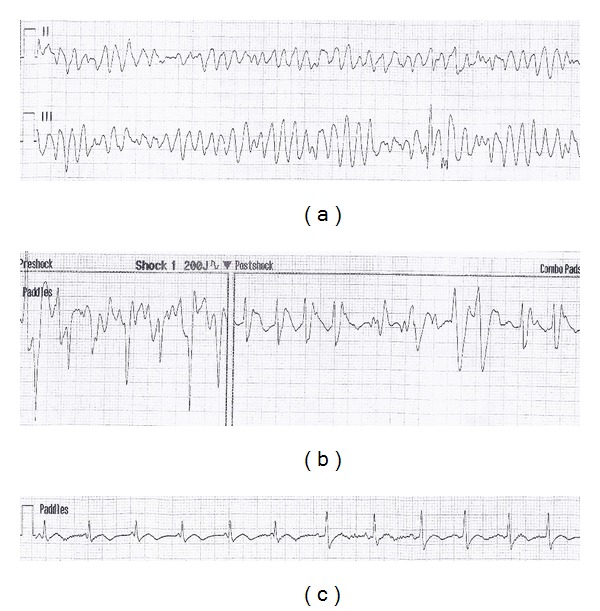
AED rhythm strip demonstrating: (a) TdP in Leads II and III, (b) resolution of TdP following defibrillation at 200 J, and (c) normal sinus rhythm in Lead II after defibrillation.

**Figure 2:**
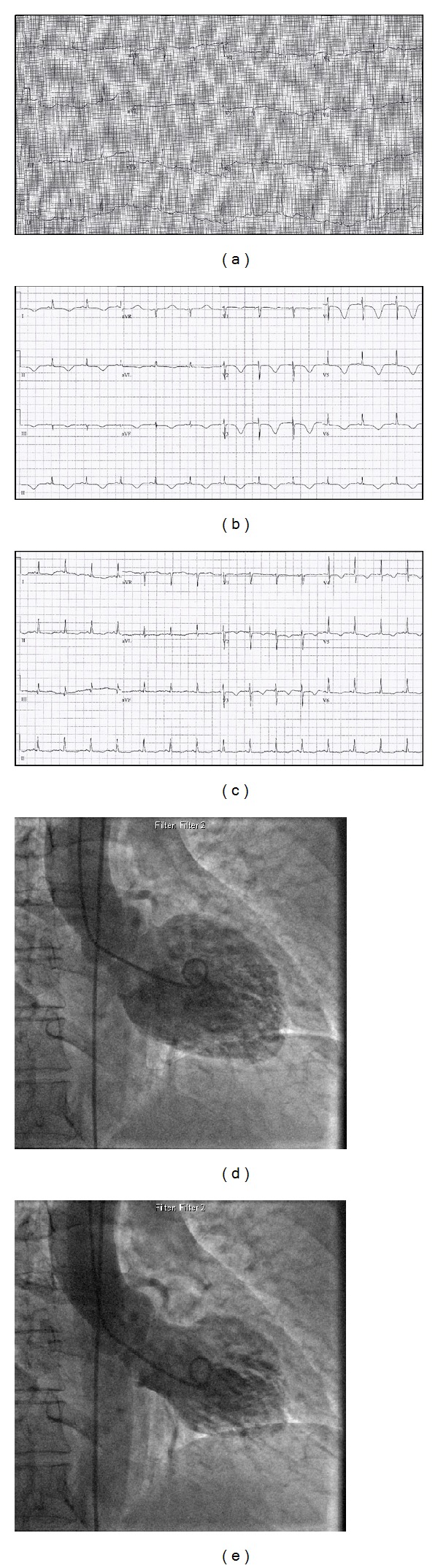
(a) ECG on admission to community hospital demonstrating normal sinus rhythm and QT interval prolongation (QTc = 544 msec). (b) ECG two days following admission demonstrating marked T-wave inversion and QT prolongation (QTc = 634 msec). (c) ECG on discharge demonstrating improvement of T-wave inversion abnormalities and shortening of the QT interval (QTc = 514 msec). (d) and (e) Left ventriculography at end-diastole and end-systole, respectively. Note the apical ballooning characteristic of Takotsubo Cardiomyopathy.

A 71-year-old woman collapsed while working as a grocery store cashier. CPR was initiated 5 minutes later and an automated external defibrillator (AED) revealed TdP. She was defibrillated and returned to sinus rhythm after a down time of 8 minutes (Figure 1). On arrival to a community hospital, her vitals included BP 120/60, HR 78, RR 20, and O_2_ saturation 99%. She was afebrile and semiconscious. Labs revealed severe hypokalemia (2.6 mmol/L) and normal cardiac enzymes. ECG demonstrated sinus rhythm with a prolonged QTc of 544 msec (Figure 2). A presumed diagnosis of ischemic polymorphic ventricular tachycardia was made and she was treated with 150 mg of amiodarone followed by infusion at 60 mg/hour. She subsequently developed hypotension with a BP of 80/60 and dopamine was administered at 15 mcg/kg/hr. Her pressure improved and potassium was given to correct hypokalemia. A temporary pacing wire was not inserted.

Her medical history included hypertension, dyslipidemia, type 2 diabetes, paroxysmal atrial fibrillation, and multinodular goiter. Home medications consisted of Amlodipine 5 mg daily, Lorazepam 0.5 mg q12h prn, Atorvastatin 40 mg daily, Citalopram 20 mg daily, Irbesartan-Hydrochlorothiazide 150 mg/12.5 mg daily, Sotalol 80 mg daily, Pantoprazole 40 mg daily, and Indomethacin 25 mg TID.

She was urgently transferred to our hospital for coronary angiography. On arrival she was nauseated and hypoxemic with an O_2_ saturation of 92% on a 100% nonrebreather. Examination revealed diffuse crackles bilaterally and a faint S1/S2 with no additional sounds. Cardiac enzymes were elevated with a troponin of 0.169 mcg/L and a chest X-ray confirmed pulmonary edema. Coronary angiogram found no significant obstructive disease in the setting of anterolateral, apical, and diaphragmatic akinesis in keeping with TCM (Figure 2). During the procedure, the patient vomited and was thought to have aspirated. Her respiratory status deteriorated and she was ultimately intubated, admitted to the ICU, and treated for congestive heart failure. Empiric treatment for aspiration pneumonia was initiated and repeat cardiac enzymes 11 hours after her collapse were elevated (troponin = 0.512 mcg/L).

Two days later, her cardiac function began to improve with mild anteroseptal hypokinesis and an LVEF of 61% measured by echocardiography. ECG findings consistent with TCM included marked T-wave inversion and QT prolongation (QTc = 634 msec) (Figure 2). She continued to improve and was extubated, and subsequent ECGs demonstrated normalization of T-wave abnormalities and shortening of the QT interval (QTc = 514 msec) (Figure 2). The patient was discharged and advised to avoid sotalol and other QT prolonging medications in the future.

## 3. Discussion

The presented case highlights the development of an acquired LQTS and TdP in the setting of TCM. Multiple risk factors for QT prolongation were present including old age, female gender, severe hypokalemia, and treatment with several QT prolonging medications. The case demonstrates the multifactorial nature of acquired LQTS and lends support to growing evidence of an association with TCM [3–6].

In this case, the patient's medications included sotalol and citalopram: two QT prolongers that delay cardiac repolarization through inhibition of the delayed rectifier potassium current (*I*_*Kr*_) [7]. These effects are compounded by severe hypokalemia, old age, and female gender making for a patient with markedly reduced repolarization reserve [8]. TCM then serves as the final insult to the myocardium, allowing for substantial QT prolongation and progression to TdP [2, 8].

In the setting of TCM, the risk of TdP escalates as QTc increases [3, 4]. This patient presented with a prolonged QTc of 544 msec that ultimately expanded to 634 msec within 48 hours. This is in keeping with classic ECG findings in TCM, whereby maximal QT prolongation and marked T-wave inversion occur 24–48 hours after onset [9]. This subacute period likely poses the highest risk for development of TdP. In spite of this, TdP occurred during the acute phase of TCM. A QTc of 544 msec supports excessive QT prolongation as the likely cause of TdP [2].

It should also be noted that the patient was treated with amiodarone despite a prolonged QTc on presentation. Amiodarone is a potential trigger for the TdP and can cause QT prolongation in the absence of TdP as well. This ultimately precipitated cardiogenic shock, highlighting the importance of identifying nonischemic causes of TdP before administering amiodarone.

Management of acquired LQTS includes withdrawal of offending medications, correction of electrolyte abnormalities, and administration of magnesium sulphate [10]. Patients progressing to TdP should receive transvenous cardiac pacing or isoproterenol infusion if hemodynamically stable [10].

## 4. Conclusion

Clinicians should maintain an awareness of the multifactorial nature of acquired LQTS. TCM should be considered a risk for acquired LQTS. Its impact is most notable during the subacute period of presentation; however, TdP can also occur in the earlier phases as well. Prompt recognition of QT prolongation is critical to ensuring safe and appropriate management.
